# The Impact of COVID-19 on Patient, Family Member, and Stakeholder Research Engagement: Insights from the PREPARE NOW Study

**DOI:** 10.1007/s11606-021-07077-w

**Published:** 2022-03-29

**Authors:** Teri Browne, Shamika Jones, Ashley N. Cabacungan, Katina Lang-Lindsey, Lana Schmidt, George Jackson, Dori Schatell, Kelli Collins Damron, Patti L. Ephraim, Felicia Hill-Briggs, Shakur Bolden, Amy Swoboda, Suzanne Ruff, Patty Danielson, Diane Littlewood, Dale Singer, Stephanie Stewart, Brandy Vinson, Diana Clynes, Jamie A. Green, Tara S. Strigo, L. Ebony Boulware

**Affiliations:** 1 College of Social Work, University of South Carolina, Columbia, SC USA; 2 Arnold School of Public Health, University of South Carolina, Columbia, SC USA; 3 Division of General Internal Medicine, Duke University School of Medicine, Durham, NC USA; 4 Department of Social Work, Psychology & Counseling, Alabama A & M University, Normal, AL USA; 5 Department of Population Health Sciences, Duke University School of Medicine, Durham, NC USA; 6 Department of Family Medicine and Community Health, Duke University School of Medicine, Durham, NC USA; 7 Center of Innovation to Accelerate Discovery and Practice Transformation (ADAPT), Durham Veterans Affairs Health Care System, Durham, NC USA; 8 Medical Education Institute, Inc., Madison, WI USA; 9 National Kidney Foundation, New York, NY USA; 10 Department of Epidemiology, Johns Hopkins Bloomberg School of Public Health, Baltimore, MD USA; 11 Welch Center for Prevention, Epidemiology and Clinical Research, Baltimore, MD USA; 12 Division of General Internal Medicine, Johns Hopkins University, Baltimore, MD USA; 13 The Care Centered Collaborative at The Pennsylvania Medical Society, Mechanicsburg, PA USA; 14 Renal Physicians Association, Rockville, MD USA; 15 Mayo Clinic Health System, Mankato, MN USA; 16 Quality Insights Renal Network 5, Richmond, VA USA; 17 American Association of Kidney Patients, Tampa, FL USA; 18 Department of Nephrology, Geisinger Commonwealth School of Medicine, Danville, PA USA; 19 Kidney Health Research Institute, Geisinger, Danville, PA USA

**Keywords:** patient-centered outcomes research, kidney disease, patient engagement, COVID-19, PCORI, patient-centered

## Abstract

**Background:**

Little is known about the impact of COVID-19 on patient, family member, and stakeholder patient-centered outcomes research engagement.

**Objective:**

To answer the research questions: (1) What is the impact of COVID-19 on the lives of patients with kidney disease and their families? (2) What is the impact of COVID-19 on research engagement for patient and family member research team members who are themselves at very high risk for poor COVID-19 outcomes? and (3) How can we help patients, family members, and stakeholder team members engage in research during COVID-19?

**Design:**

We conducted virtual semi-structured interviews with patient and family member co-investigators and kidney disease stakeholders from the PREPARE NOW study during November 2020. The interview guide included questions about participants’ experiences with the impact of COVID-19 on research engagement.

**Participants:**

Seven patient and family member co-investigators and eight kidney disease stakeholders involved in a kidney disease patient-centered outcomes research project participated in the interviews, data analysis, and writing this manuscript.

**Approach:**

We used a content analysis approach and identified the main themes using an inductive process.

**Key Results:**

Respondents reported three main ways that COVID-19 has impacted their lives: emotional impact, changing behaviors, and changes in health care delivery. The majority of respondents reported no negative impact of COVID-19 on their ability to engage in this research project. Suggestions for patient-centered outcomes research during COVID-19 and other emergencies include virtual research activities; active engagement; and promoting trust, honesty, transparency, and authenticity.

**Conclusions:**

COVID-19 has had a significant negative impact on patient, family member, and stakeholder research team members; however, this has not resulted in less research engagement.

**Trial Registration:**

Clinicaltrials.gov NCT02722382

**Supplementary Information:**

The online version contains supplementary material available at 10.1007/s11606-021-07077-w.

## BACKGROUND

Patient-centered outcomes research (PCOR) is essential for testing new interventions for chronic illnesses such as kidney disease and examining their impact. Kidney disease PCOR has been growing internationally.^1–9^ One example is the PREPARE NOW study, a 5-year cluster randomized controlled trial examining the effect of systemic changes on kidney disease care.^10–13^ This study, funded by the Patient-Centered Outcomes Research Institute (PCORI), includes people with kidney disease and family members of people with kidney disease as study co-investigators and kidney organization stakeholders as research team members.

The COVID-19 pandemic has had a significant impact on the kidney disease community at large in the USA. People with kidney failure are among the highest-risk groups for severe COVID-19 infection^14^ and poor COVID-19 outcomes.^14–16^ Compared to people with COVID-19 in the general population, patients with kidney failure and COVID-19 are at a greater risk for rapid progression of the disease^15^ and increased mortality.^17–21^

While there have been recommendations on the conduct of PCOR and other clinical research during COVID-19,^22–24^ little is known about the impact of COVID-19 on the ability of patients, family members, and stakeholder organizations to maintain active participation in PCOR engagement. Given that COVID-19 will have a global impact for the foreseeable future, it is crucial to understand how this pandemic is impacting PCOR. Social distancing, travel restrictions, and other safety measures required to minimize transmission of COVID-19 may have deleterious effects on PCOR engagement. This is particularly likely among populations at the highest risk for poor COVID-19 outcomes, such as people with kidney failure. People living with kidney disease have recounted disruptions in daily routines and medical care, social isolation, and psychosocial impacts.^25^ As a result, patients, family members, and stakeholders may not be able to engage in research as they did before COVID-19.

The importance of what we have learned about patient engagement in research goes beyond the immediate COVID-19 pandemic. Much as telemedicine became rapidly more common at the beginning of the COID-19 pandemic,^26^ the use of alternative workflows for PCOR studies occurred almost overnight, including alternative meeting platforms and interactions that had to be fit into broadly changing life circumstances. Just as health systems are currently planning for post-pandemic systems for telehealth services, PCOR researchers must prepare for how engagement should best occur in post-pandemic projects.

To explore the impact of COVID-19 on research engagement by patients, family members, and stakeholders in the PREPARE NOW study, we conducted qualitative interviews to answer the research questions: (1) What is the impact of COVID-19 on the lives of patients with kidney disease and their families? (2) What is the impact of COVID-19 on research engagement for patient and family member research team members who are themselves at very high risk for poor COVID-19 outcomes? and (3) How can we help patients, family members, and stakeholder team members engage in research during COVID-19? Lessons learned from this study can provide insight on how to help patients, family members, and stakeholders engage in research during COVID-19 or other emergencies.

## METHODS

### Study Design and Participants

In November 2020, we conducted a qualitative study to assess how COVID-19 has impacted patient, family member, and stakeholder engagement in the PREPARE NOW research study. Described previously,^13^ PREPARE NOW (NCT 02722382) is a cluster randomized controlled trial examining the effectiveness of a multi-faceted intervention to improve shared and informed decision-making in the care of patients with kidney disease.

Six patient and two family co-investigators and eight representatives from eight kidney disease stakeholder organizations (American Association of Kidney Patients; Council of Nephrology Social Workers; Geisinger Health System; Medical Education Institute, Inc.; National Kidney Foundation; Quality Insights Renal Network 5; Renal Physicians Association; The Care Centered Collaborative at The Pennsylvania Medical Society) have been active research partners from the time of project inception in 2013.^12^

As previously described,^12^ patient and family co-investigators have been very involved with the study from the project’s conception to dissemination. Over the 4 years of the project before the COVID-19 pandemic, co-investigators participated in regular meetings with research team leaders and staff (usually monthly), where they reviewed updates on the project and provided input into all aspects of the research. Representatives from stakeholder organizations provided advice on the project at quarterly full team meetings. Co-investigators and stakeholders were also members of project workgroups (i.e., recruitment, data collection, intervention design, data analysis). Patient and family member co-investigators were asked to participate in these interviews at a meeting, and through email, and stakeholders were invited to participate by email. Each interviewee participated in one interview, and there were no repeat interviews. To maximize data saturation of project representatives, every patient and family co-investigator and stakeholder was invited to participate in an interview.

There was no additional compensation for participating in these interviews. Interviewees were compensated annually for their work on the PREPARE NOW project, which included time to participate in these interviews. The institutional review boards of Duke University (Pro00074588) and the University of South Carolina (Pro00104964) approved this study. We used the consolidated criteria for reporting qualitative research (COREQ) in this study’s implementation and reporting.^27^

### Semi-structured Interviews

One semi-structured interview guide was used to conduct the patient and family co-investigator interviews, and one guide was used for the kidney disease organization stakeholders (Appendices A and B in the Supplementary Information). Both of these interview guides were co-created by the patient and family member co-investigators, aligned with the ethos of PCOR.^28^ This approach (of the interviewees co-creating the interview guides) allowed for maximum co-construction of knowledge and co-ownership of the research, central tenets of community-based participatory research^29, 30^ (including PCOR).^31^ The first step in designing the interview guides was reviewing the PCOR engagement literature and the PCORI Engagement Tool and Resource Repository.^31^ Interview questions were guided by this literature search and study research questions and included questions from the *Ways of Engaging-Engagement Activity* tool.^32^ These questions included prompts about the level of trust, honesty, transparency, and authenticity participants felt while working on the project—central PCOR principles.^28, 32^ Patient and family co-investigators reviewed draft interview guides independently and as a group during a virtual meeting. The interview guides were refined to incorporate their feedback and suggestions. Interviews were completed by a female public health researcher with qualitative research training and experience and who had no prior relationship to the participants or the PREPARE NOW project (author S.J.). Interviewees were informed about the interviewer’s background, and the interviewer had previous experience with kidney disease research. Interviews were conducted using Zoom video conferencing software,^33^ and the interviewer and interviewees were the only ones present during the interviews. Virtual interviews were required due to the broad geographic locations of the participants, travel restrictions, and the need to isolate for COVID-19 safety. The interviewer kept field notes on each interview.

Informed consent was verbally reviewed with each participant at the beginning of each interview, and consent was obtained for the interviews, recordings, and transcripts. All interviewees were assigned an identifier code so that recordings and transcriptions were de-identified to increase privacy. Each interview was recorded using Zoom’s recording function and transcribed verbatim by a transcription service. The interviews lasted between 30 and 73 min (M=49 min, SD=14).

### Analysis

The interview transcripts and field notes were analyzed using MaxQDA 2020 software. Two experienced qualitative researchers (T.B. and S.J.) conducted the analysis using content analysis.^34–36^ The first cycle of line-by-line coding of all transcripts was performed by both authors sequentially using provisional codes, and code memos derived from interview prompts. New codes that emerged from the data were added. The second cycle of coding was performed using axial coding to differentiate and organize sub-codes used to identify the most salient themes.^37^ These two authors discussed final codes and themes and finalized the coding structure based on consensus. All authors (including all interviewees except for one patient co-investigator who passed away before data analysis) reviewed and provided feedback on the results and themes were finalized. The dataset analyzed during the current study is available from the corresponding author on reasonable request.

## RESULTS

A total of 15 participants were interviewed: seven patient and family member co-investigators and eight representatives from kidney disease organization stakeholders. Among the co-investigator interviewees, two were family members of people with kidney failure, and five were people with kidney failure. The patient co-investigators had experience with all forms of kidney failure treatment—in-center hemodialysis, home dialysis, and kidney transplant. The family co-investigators were the wife of a patient who received a kidney transplant and a kidney living donor with many family members with kidney disease. All of those who were invited participated in the interviews, except one patient who was unable to do so because he was hospitalized. All interviewees except for one stakeholder had worked on the PREPARE NOW project since it began in 2013. The results are described in three themes that align with the study research questions: (1) COVID-19 impact on life in general, (2) COVID-19 impact on research engagement, (3) suggestions for PCOR during COVID-19. Themes, subthemes, and supporting quotes are summarized in Table 1.

**Table 1:** Themes, Subthemes, and Supporting Quotes

**Theme**	**Subtheme**	**Supporting quotes**
1. COVID-19 impact on life in general	1a. Emotional impact of COVID-19	I had to go with my husband…for his checkup and that was terrorizing (Co-I 1) I feel like I am shut off from the world… It gets lonely. You want to see people… I am very, very, very, very cautious at this point because I know that if anything, if I contact this, the chances of my mortality will be, is higher. (Co-I 7) It’s just the worst time that we’ve experienced. (Stakeholder 4) They [patients] especially do not want to go into the clinic when there is a global pandemic happening and they are at higher risk than everybody else… These are very scary times for a lot of people. (Stakeholder 5) There was the fear of the unknown. A lot of mental and coping issues kind of came about with the self-isolation and the lockdowns that occurred earlier in the pandemic. You know, a lot of patients were having health issues because of a fear of going to dialysis appointments or going to doctor’s appointments. (Stakeholder 7)
	1b. Changing behaviors because of COVID-19	Our lives outside of our homes have stopped pretty much (Co-I 1) We don’t go anywhere anymore (Co-I 4) I certainly have had a lot of thought about what could happen if I get COVID, and I actually planned my funeral (Co-I 6)
	1c. Changes in health care engagement	I still feel really uncomfortable going to the dentist. I know we have to go, but it freaks me out… we both haven’t gone to the dentist so we’re overdue for six month follow ups. My regular well woman checkup has been delayed and no one’s really followed up with me to reschedule it. They cancelled it but they never rescheduled it. I’m not pushing it…right in the beginning I was like we’re going to miss appointments that we had in person until virtual visits took place. (Co-I 2) I try to tell them to give me the earliest appointment possible. So that has been a change right there. I tell them I want the earliest appointment available, so if I say I want the eight o’clock appointment when they first opened. Okay. So that’s one change that I have been trying to get the earliest appointments possible so that I can be in and out and I have to wait. (Co-I 3) I didn’t really want to go to the doctor’s office because those are probably the worst places to go. (Co-I 5) My ENT, my ear, nose, and throat was delayed because that wasn’t urgent, and I wasn’t having any problems. (Co-I 6) I think one thing is it changed the way that we do our work, right? And meaning – what that means is, you know, in the beginning patients weren’t able to come in for their visits. I think patients were a little leery on seeking healthcare. Now what we’ve noticed in this round it’s a little different, right? So we go back to that when we first started this COVID journey back in March, right? People weren’t seeking healthcare at all, right? They were scared to go to E.R.s, they were scared to go to doctors’ offices. You know, even if they were open, right? And we didn’t have a lot of conversations with those patients. Now did we have our outreach to patients, you know, we did our best to keep that moving. Now the second round seems a little bit different. We’re seeing more people going to the E.R. with chronic conditions and in the patients that have COVID - even if they’re doing okay, I think they’re scared and they’re seeking care a little bit earlier, right? So I think that makes things a little bit more crazy and that’s one of the things we’re experiencing right now. (Stakeholder 6) A lot of people die on dialysis but a lot of people die because they had symptoms and they’re like, no, I’m not going to that ER. I’m not going to that urgent care. I’m not going to the doctor. It’s not safe out there. But the reality is, and it was scary, even for me, it was scary to go in and get healthcare, but they’re being very, very careful at every healthcare setting I’ve been to. (Stakeholder 25) At the very beginning of COVID I would say we did hear quite frequently of a lot of patients missing appointments or maybe having an issue not going to their doctor, not going to the hospital, and then it ends up kind of compounding and being more severe than if they would’ve thought, you know, treatment early or so. (Stakeholder 27)
2. COVID-19 impact on research engagement	2a. Negative impact on research engagement	The only big thing is that we typically would meet every year in person, and now that’s virtual, which is kind of, it’s unfortunate because I really liked seeing everyone once a year. We have a lot of great stakeholders, and it’s just really wonderful. They’re kind of almost like an extended family, so it’s nice to see them and catch up and everything like that. So, that’s disappointing. (Co-I 2) I just feel like there’s been so many other things happening and quick responses to things that have needed to occur in order to keep our organization afloat that the PREPARE NOW project just hasn’t been a priority. (Stakeholder 2)
	2b. No or positive impact on research engagement	No stress at all about being a part of it. And strongly agree and recommend being a part of it during COVID-19. It keeps you busy. It occupies your mind. And it helps you focus on work that needs to be done for when COVID is all gone. (Co-I 1) I personally feel from a patient organization it adds hope and it keeps us on the right direction because, as I said earlier in the conversation, you know, all the issues that kidney patients normally face or have to contend with don’t just go away because of COVID. So, I think it’s important and respectful and necessary to continue research. So individuals that are impacted by kidney disease understand that, you know, even though the world may be in kind of a chaos with this pandemic, that there are still people out there that are striving for research and innovation and improving patient care. And, you know, COVID actually just kind of put a spotlight on some issues that were already in the kidney community with health inequities, some disparity issues and things of that nature. So, if anything, I think it kind of gave us a catalyst to improve maybe the work that we’re already doing. So, I actually, I view it as a positive to keep moving forward with research (Stakeholder 7) I like it because it doesn’t talk about COVID-19. So, it’s kind of a break. It’s a welcome distraction… Everybody talks about the COVID fatigue, and this is a way to energize again. (Stakeholder 8)
3. Suggestions for PCOR during COVID-19	3a. Virtual research activities	What’s nice about this project is that they’ve always done virtual conferencing. And so, the nice thing is that wasn’t anything new for us. (Co-I 2) I really enjoy the virtual meetings because I can just do it from my home. I don’t have to move or stand or pack a bag or catch a flight or anything. (Co-I 5) Getting people used to technology is important. (Stakeholder 6)
	3b. Actively engage patients and family members in research	The way the study was even started, I mean including all of us and every aspect of the research study just shows the trust, right, the level of trust and transparency. There was nothing, no area that we couldn’t be involved in. So, it was all that, all of those things just kind of fell into place. But I think that’s honestly inherent to the people who are running the study and everyone involved, the coordinators, the research leads. No one ever kept us in the dark about anything…The coordinators of the projects were excellent. And if they couldn’t get a hold of us, they would email us, would call us if needed. And they were always very on the ball and really active with us. So, I think having really strong coordinators for projects is incredibly key. I mean they were the lifelines…They were always open to hearing everyone’s opinions and personal stories and suggestions. I mean they just made a very safe and comfortable environment to bring any suggestions, and they took everyone very seriously and followed through with any questions that people had. (Co-I 2) They are excellent on valuing everyone’s input. They’re not dismissive of anyone’s input. And they listen to the patients. Of course, patients, we’re all usually shoved to the side. But they really want to hear from us. (Co-I 5) I feel like we know each other for life now… When it would come to a standstill, or where it comes to a block in the road in their recruitment, they’ve reached out to us and asked us, what do you think about this, what are your insights on this? And I think that right there has been, it’s been very, very, very –it’s made, I know for me, it makes me as a patient being involved in this research study, it made me feel valued. (Co-I 7) I think most patients, particularly during COVID, were happy to have any sort of outreach, whether it’s a clinical person checking up on them, or as part of a research study. I think people are feeling isolated, and so those types of outreach, in whatever format they came, would be welcomed by many folks. (Stakeholder 3) I think it was the very early engagement, the communication, their transparency. And as we did this, they were listening. They listened, and if we said something, they took our advice or just heard our voices, and could help with improving something as we moved ahead building the Prepare Now study. (Stakeholder 4)
	3c. Promote trust, honesty, transparency, and authenticity in PCOR	It’s the most wonderful way to feel like you have some control over your disease, or whatever issues you’re battling…It gives me a feeling of accomplishment and helps me feel less frustrated by the disease that my family has. (Co-I 1) They listened to us and then modified that process based on input that we provided. (Co-I 3) Our value to the research project was always honored. (Co-I 6) I think everybody’s voices were listened to. I think that there was a real receptivity to hearing from all of the stakeholders. (Stakeholder 2) There was communication, there was transparency, there was a partnership of friendship, engagement, really working together. I think that’s the foundation for everything. (Stakeholder 4) Everybody made you feel like you were family, like they knew you very well. And I think the communication style, it was very friendly, supportive, encouraging. (Stakeholder 8)

### COVID-19 Impact on Life in General

COVID-19 has significantly impacted the everyday lives of all PREPARE NOW patient and family member co-investigators [Theme 1 in Table 1]. Project stakeholders discussed the pandemic’s impact on their lives and the impact on patients, family members, and health professionals. Sub-themes for this theme include the emotional impact of COVID-19, changing behaviors, and changes in health care engagement.

#### Emotional Impact of COVID-19

All patients and family members described negative emotions related to COVID-19 [Subtheme 1a]. Stakeholders also described how patients and health care professionals they work with are negatively impacted by COVID-19. The most common reactions were fear, anxiety, and stress in response to their own or their family members’ susceptibility to getting COVID-19 and having poor outcomes.

#### Changing Behaviors Because of COVID-19

All interviewees discussed several things they are doing differently because of COVID-19 to keep themselves and their families safe during the pandemic [Subtheme 1b]. The main behavior changes were wearing masks, staying home, working from home, avoiding crowds, homeschooling, and not seeing people outside of their households. Two co-investigators reported significant anticipatory behaviors in case they or someone they live with gets COVID-19. One patient reported that she had planned her funeral, and one family member had made plans for childcare if her spouse gets COVID-19.

#### Changes in Health Care Engagement

All interviewees mentioned changes they have experienced or witnessed in health care engagement [Subtheme 1c]. Almost all co-investigators used telehealth for the first time since the pandemic started for nephrology and other appointments. Five of the seven patients reported missing or delaying medical appointments because they did not feel safe going out in public. This included delaying dental care and laboratory appointments. Stakeholders also discussed how patients their organizations worked with missed medical appointments because of COVID-19.

Participants were asked what health care providers can do to make them feel better about going to medical appointments during COVID-19. One patient reported, “I’m sorry, they’re not going to be able to. I’m a healthcare worker. They’re going to have to seriously do some major convincing, and I don’t know if they can enough.” Several participants relied on different strategies to minimize their risk when attending in-person medical appointments. One family member co-investigator reported that she and her husband drove from North Carolina to Ohio for an out-of-state medical appointment instead of flying to minimize their exposure to COVID-19 and taking the earliest possible appointments available at their providers when it is less busy. 

### COVID-19 Impact on Research Engagement

Interviewees reported some negative consequences of the pandemic on their ability to engage in research [Theme 2 in Table 1]. However, all patients and family members and many stakeholders did not feel that COVID-19 had a significant impact on their research engagement. Some interviewees reported that COVID-19 enhanced their ability to participate as research team members.

#### Negative Impact on Research Engagement

The primary way that COVID-19 impacted the interviewees’ ability to engage in research was the cancelation of a 2020 in-person team meeting [Subtheme 2a]. Every co-investigator and stakeholder mentioned this cancelation and missed being able to see each other in person. Co-investigators who had attended the national PCORI meeting in previous years also said they missed going to that event in person.

Some of the kidney disease organization stakeholders (*n*=3), especially those who worked for health systems or in direct patient care, did report some changes in their ability to engage in this project. Attending to COVID-19 in their organizations required much of their time in 2020, and they did not have the time they usually did to participate in research collaboration.

#### No or Positive Impact on Research Engagement

Despite missing the canceled in-person research team meeting, all co-investigators and five stakeholders reported minimal disruption in their ability to engage in research because the project was already conducting most of its engagement virtually [Subtheme 2b]. Project work during the pandemic continued as it did before COVID-19—by telephone or webinar meetings.

Six interviewees thought that they were able to better engage in research because of COVID-19. This was because this work gave these team members hope or a distraction during a difficult time. Some respondents reported that they had more time to devote to research as they were not spending as much of their time traveling or commuting as they usually did. Despite a few challenges engaging in research during COVID-19, all interviewees said they would participate in another similar research project in the future.

### Suggestions for PCOR During COVID-19

Overall, all participants had a generally positive experience with research engagement on the PREPARE NOW project, including during COVID-19. Some suggestions for improving patient, family member, and stakeholder engagement in PCOR during COVID-19 include virtual research activity; active efforts to engage these research team members; and promoting trust, honesty, and transparency [Theme 3 in Table 1].

#### Virtual Research Activities

All the interviewees appreciated virtual research meetings during COVID-19 [Subtheme 3a]. Co-investigators and stakeholders mentioned the need to have both telephone and computer access to such meetings to maximize patient and family member participation. Interviewees shared that they believed that older adults especially may have difficulty accessing virtual meetings. They discussed that patients and family members may not have internet access, or their phones may not allow for videoconferencing. Therefore, having a phone option for virtual meetings is necessary. A few patients discussed how they participate in meetings by telephone only, and they did not have data plans that allowed for videoconferencing on their phones. Some other suggestions related to virtual research activities include using large fonts for all virtual presentations, providing patients and family members with laptops, and having technology assistance available for everyone. Additional suggestions include contacting people by phone in addition to email, providing people with simple instructions about how to use virtual meeting platforms, and including clear explanations about if a waiting room or password will be used.

Despite all interviewees’ appreciation for virtual project work, two stakeholders cautioned about virtual meeting fatigue, with one reporting being “Zoomed out.”

#### Actively Engage Patients and Family Members in Research

The co-investigators provided multiple examples of ways that the PREPARE NOW project successfully engaged them in PCOR, which continued during COVID-19 [Subtheme 3b]. Project leaders and staff consistently made sure that these co-investigators played a vital role in the project and frequently communicated with them.

All patients and family members enjoyed the small group meetings that were held for the co-investigators each month. They discussed how project leaders actively asked for their input during all meetings and gave everyone a chance to participate and contribute. Having a designated project point person for the patient and family member co-investigators was mentioned as helpful by six interviewees. Patients and family members enjoyed the other PCOR opportunities they were informed about by project leaders and staff beyond the PREPARE NOW project. Several of the co-investigators were active PCORI ambassadors and involved in other PCOR projects because of their work in this project.

#### Promote Trust, Honesty, Transparency, and Authenticity in PCOR

Like the active project engagement that continued during COVID-19, interviewees also discussed ways that the team leadership promoted trust, honesty, transparency, and authenticity in PREPARE NOW and their favorable experiences with these PCOR principles in this study [Subtheme 3c]. Interviewees were asked, “How much did you feel trust, honesty, transparency, shared-learning, and give-and-take relationships while working on this project?” All co-investigators and all but one stakeholder reported “a great deal” in response to this question (one stakeholder answered “somewhat”).

These PCOR standards helped interviewees feel engaged in this project even during COVID-19. Team leaders promoted these PCOR values by “authentically listening” to these team members and using their feedback provided in the project. For example, patients and family members recalled instances of suggesting ways to improve study enrollment and those ideas being used in recruitment materials.

Co-investigators also felt that project leaders were genuine with them, which was exemplified in several ways. Co-investigators talked about how it was important that project leaders create a space for the team to get to know each other personally. For example, they talked about how at the beginning of the project, every team member did a brief biography that included personal details about them (see https://www.kidneypreparenow.org/our-team.html). Several respondents talked about now being friends with the other research team members due to their work together on PREPARE NOW. Project leaders also regularly checked in with the co-investigators and talked about their lives, families, and personal milestones or celebrations during monthly meetings. These efforts to encourage engagement continued after COVID-19. In 2020, there was a meeting to discuss the pandemic’s impact on the co-investigators. All interviewees attributed frequent communication to continued project engagement during COVID-19.

Additionally, participants reported that they felt valued throughout the study. Most participants reported feeling like they made a meaningful impact, but a few stakeholders were unsure if their impact was meaningful, suggesting better communication was needed with stakeholders. Three stakeholders also suggested establishing clear roles and expectations early in the project. Having co-investigators and stakeholders connect and share their inspirations for participating in the project helped engage them in the research, despite the pandemic.

## DISCUSSION

These results suggest that COVID-19 has had significant and negative impacts on people with kidney disease and their family members. Despite this, all patient and family member co-investigators and most stakeholders remained as engaged in the PREPARE NOW study as they were before COVID-19. Some interviewees were more willing to engage in research during the pandemic, as the work gave them hope or a distraction from COVID-19 or had more time available. Study leadership needs to ensure that they put systems in place to actively engage patients, family members, and stakeholders and earn their trust.

This study supports other findings of the psychological distress of COVID-19^38^ and confirms the impact of COVID-19 on family members of people with chronic illnesses.^39, 40^ Researchers doing PCOR during COVID-19 are encouraged to acknowledge and discuss the emotional impact that COVID-19 has had on its team members. Some other insights from this study are the importance of virtual meetings. While the pandemic has created a multitude of difficulties, particularly for vulnerable populations, it has also exposed areas of opportunity, particularly in engaging patients in research. By and large, engaging patients in research has not posed new challenges during the pandemic for our study. While we recognize this may not hold true for all research, it has highlighted the need for increased attention to both technologies to better mimic in-person experiences and to assess and try to address patient stakeholders’ technological barriers. Researchers may want to have conversations with patients and family members at the beginning of projects to evaluate their access to electronic devices, high-speed internet, and comfort in using technology. Researchers also may want to offer virtual meetings that are accessed both by video and telephone. Funders are encouraged to cover the cost of “loaner” laptops, portable internet hotspots, and technical support for patients and family members who need such assistance to participate in PCOR.

For several in our study, being at home has increased their ability to participate in research. If remote patient, family, and stakeholder input can be optimized to create the same level of engagement that we have seen in our in-person meeting, this would revolutionize the way we can engage patients and family members in research and allow those to participate for who travel was previously a deterrent to participating.

Researchers leading or interested in doing PCOR projects can use these findings to maximize authentic engagement with patients, families, and stakeholders during regional, national, and global emergencies. The PCORI website also has multiple resources for investigators to improve trust, honesty, transparency, and authenticity in this engagement (www.pcori.org). This study is important because it is the first to examine the impact of COVID-19 on patient, family member, and stakeholder research engagement. There is a need for PCOR to continue during COVID-19 or any future pandemic or disaster. Emerging research suggests that having a PCOR team in place may facilitate examining the pandemic’s impact on patient communities.^41^ Some suggest that COVID-19 offers an opportunity to re-design health care to be more patient-centered,^42^ and PCORI has multiple studies currently examining the impact of COVID-19 on research ^43^.

The findings also provide important considerations for a post-COVID-19 research environment. Virtual meetings offer a way to interact with patient and family member co-investigators and stakeholders effectively. However, if individuals do not have access to necessary technology and internet access (and the ability to use them), they may not feel fully integrated into the research. This is a crucial consideration for studies seeking to gain a full range of needed perspectives.

There are a few limitations of this study. Although we interviewed almost every patient and family co-investigator (except for one who was hospitalized) and every stakeholder in this project to maximize data saturation, the findings may not be similar to other kidney disease patient, family member, and stakeholder research partners. Our project also was in its final year when COVID-19 started, so the team had already coalesced before the pandemic. Other PCOR projects may not have a similar established engagement of patients, family members, and stakeholders before a disruptive event. The patient and family co-investigators in our study had consistently been very active in research prior to COVID-19, and the impact of the pandemic on their research engagement may not be a common experience. This study also relates to a kidney disease project, and our findings may not apply to other diseases or treatments. We also had been using virtual meetings before COVID-19, so patients, family members, and stakeholders on projects utilizing that form of engagement for the first time may have different experiences. Projects that have not previously used virtual engagement will quickly need to create strategies to assess patient, family member, and stakeholder access to the internet and technology and develop solutions for any barriers.

Further research is needed to examine the impact of COVID-19 on PCOR research engagement with other populations and other kidney disease studies. This research should include both patient and family member research partners and organizational stakeholders. Although these groups had many common answers in this study, there were some different responses and concerns.

Research studies during a pandemic are essential, desired by the public,^44^ and require careful ethical considerations.^45^ Before the COVID-19 pandemic, there was a global clarion call to involve patients, family members, and professional stakeholders as research partners to improve health outcomes. We know that COVID-19 has required people, especially those at high risk for poor outcomes, to social distance and remain home as much as possible, and this likely will continue for the foreseeable future. Exemplars must be identified to continue patient-centered outcomes research efforts seamlessly, whether remote or in-person. Lessons learned from this pandemic may be transferable to patient-centered outcomes research during future regional, national, or global emergencies.

## Supplementary Information

ESM 1 (DOCX 30 kb)

## Declarations

### Disclaimer

The views expressed in this paper are those of the authors and do not reflect the position or policy of the Department of Veterans Affairs or the US government.
