# Supplementary material for: The Impact of COVID-19 on Patient, Family Member, and Stakeholder Research Engagement: Insights from the PREPARE NOW Study
Source: J Gen Intern Med. 2022 Mar 29;37(Suppl 1):64–72. doi: 10.1007/s11606-021-07077-w (PMC8960674; doi:10.1007/s11606-021-07077-w)
Supplement: Supplementary file 1 — (DOCX 30 kb) [file 11606_2021_7077_MOESM1_ESM.docx]

**Appendices**

**Appendix A. Patient and** **Family Member Co-Investigator Interview Guide**

**FILL OUT FOR EACH INTERVIEW:**

**Interview with (check and circle one, can cross check interviewee name with the list in Trello)**

- **Patient or family member Co-Investigator**
- **Kidney disease stakeholder**

**Assign the interview an identifier code:**

i.e. Co-I #1 (1-7), Stakeholder #1 (20-27)

**Hello! This is NAME calling from the PCORI PREPARE NOW study. Thanks so much for agreeing to talk to me today. We want to learn about how COVID-19 has impacted your life and also your ability to engage in our PCORI PREPARE NOW study. We would like to ask you some questions that should take less than an hour to complete.**

**Is this still a convenient time for you to talk?**

**If No**

No problem, we will reach out to you by email to schedule a better time to talk

*Email NAME and work with her to reschedule the interview*

**If Yes**

Great! I really appreciate your time today. I am going to go ahead and ask you some questions. Before I do, I want to assure you that what you share with me will be kept anonymous. I will record this interview just so I can be sure I don’t miss anything that you are saying- and the recording and transcript of our interview will be kept in protected and secure files. I am not going to ask your name, so your name will not be on the transcript or recording, and the information you give us will be combined with what other people tell us, so we will not be able to know who said what. We just want to talk to the people involved in the study to see how we did in engaging you in the research process, what are some good things we did, how we can make improvements- and also share lessons learned about how we can help patients, family members and stakeholders take part in research during COVID-19. If you have any questions, you can contact NAME at the University of South Carolina.

**Do you have any questions about anything I just said? Were there any parts that seemed unclear?**

**1. Do you agree to do this interview?**

- Yes
- No

**2. Do I have your permission to record this interview?**

- Yes
- No

**If no consent  Thank you for your time. Have a wonderful day.**

*****END INTERVIEW*****

**IF YES:** Thank you!

1. First, can you tell me- on this PREPARE NOW study team, were you a patient or family member Co-Investigator, or were you a kidney disease organization stakeholder?
2. Great- I am going to start off by asking- in general, how has COVID-19 impacted your life?

Probe -- Personally? Professionally?

1. As someone who is high-risk for bad things to happen if you get COVID-19, or a family member of someone at high-risk, do you think you have had to do things differently than other people who are in good health (not high-risk)?
   1. **Yes**
      1. What things have you had to do differently in your life because of COVID-19?
   2. **No**
      1. Great, thanks- what sorts of things have you been doing to be safe during COVID-19?
2. Thanks for sharing that. As you know, you have been part of the PCORI funded PREPARE NOW research study for the last five years as a Co-Investigator. Can you think back starting earlier this year when COVID-19 started in the United States- until now- can you share with me if COVID-19 has made a difference (impacted/had an effect) in your participation and engagement with the PREPARE NOW project?
   1. (PROMPT-For example, were you unable to participate in meetings or read emails or information for this project because of COVID-19?)
   2. LET THEM ANSWER- THEN-
   3. **If YES effect**
      1. Thanks- can you tell me more about this
         1. LET THEM ANSWER- THEN
            1. **As you know, almost all of the PREPARE NOW project work you have done is done virtually- by phone, webinar or email**. **Has that been helpful for you to participate in the PREPARE NOW project during this year when COVID-19 was impacting everything?**

If yes- how so? Can you tell me more about that?

If no- thanks, can you tell me more about what you mean?

- 1. **If NO effect**
     1. Thanks- you started off mentioning some ways that COVID-19 has impacted your and your family’s life- why do you think that it hasn’t impacted how you are able to participate and engage in the PREPARE NOW project?
        1. LET THEM ANSWER- THEN
           1. **As you know, almost all of the PREPARE NOW project work you have done is done virtually- by phone, webinar or email**. Has that been helpful for you to participate in the PREPARE NOW project during this year when COVID-19 was impacting everything?

If yes- how so? Can you tell me more about that?

If no- thanks, can you tell me more about what you mean?

1. Thanks so much. This is great information. Let me ask you- what are some ideas you have for how the PREPARE NOW team (or other research projects) can best help patient and family team members to fully engage in and be part of research projects while at the same time they are dealing with COVID-19’s impact on themselves and their families?
   1. Probe for specific suggestions- can also restate: We know that COVID-19 is going to be around for a while, what are the best ways for researchers to partner with patients and family members in research during COVID-19?
      1. LET THEM ANSWER- THEN
         1. As you know, almost all of the PREPARE NOW project work you have done is done virtually, or remotely- by phone, webinar or email. What might be some issues or problems with that for other patients and family members working with different research projects you can think of? Why might that be a problem?
            1. LET THEM ANSWER- THEN

Thanks, what are some things that our project has done well to keep you engaged and active, that we can implement on other research projects where we can involve stakeholders on research teams during COVID-19?

1. What has your experience been (what has it been like for you) with our virtual form of research engagement? (work done remotely, through emails, calls, and webinar meetings)
   1. Probe for specific suggestions
      1. Do you like doing work this way?
      2. What are the best things about it?
      3. What don’t you like about it?
      4. What would you like done differently?
2. Overall, how satisfied and engaged have you felt working on the PREPARE NOW project as a Co-Investigator?
   1. LET THEM ANSWER- THEN
      1. Has this changed- how satisfied and engaged you have felt- before and after COVID-19?
         1. If **NO**
            1. Thanks, why do you think you haven’t felt any differently about how satisfied you are with your work as a Co-Investigator on the PREPARE NOW project? can you describe ways your engagement on this project has NOT been affected by COVID-19?
            2. Additional probes- **some people may feel that being part of a research project during COVID-19 could offer hope or positivity in this difficult time, or some others may feel it adds additional stress. Have you felt this way being part of PREPARE NOW or any other research projects during COVID-19?**
         2. If **YES**
            1. Thanks, please tell me a little bit more about how COVID-19 changed the way you feel satisfied and engaged with your work as a Co-Investigator on the PREPARE NOW project? - can you describe ways your engagement on this project has been affected by COVID-19?

Additional probes- **some people may feel that being part of a research project during COVID-19 could offer hope or positivity in this difficult time, or some others may feel it adds additional stress. Have you felt this way being part of PREPARE NOW or any other research projects during COVID-19?**

1. Have you (or your family member) missed any medical appointments, or not getting any bloodwork or medical testing done, because of COVID-19?
   1. If YES
      1. Probe- can you tell me more about that?
         1. What appointments did you (or family member) miss?
         2. Why did you (or family member) miss the appointments? (COVID-19 related)
         3. What do you think doctors and healthcare providers can do that would make you (or family member) feel better about going to medical appointments during COVID-19?
            1. What can they do to help you (or family member) feel safer?
   2. If NO
      1. Probe- can you tell me more about that?
         1. Did you (or family member) have medical appointments to go to since COVID-19 started?
            1. If YES

Can you tell me what kind of appointments?

Can you tell me about your (or family member) decision to keep these appointments?

1. Have you (or family member) used telehealth or remote medical visits (by phone, or the internet) because of COVID-19?
   1. If YES
      1. Probe- can you tell me more about that?
         1. What appointments did you (or family member) have that used telehealth?
         2. What was your experience using telehealth or remote visits?
            1. Did you (or family member) like it? not like it? what was good/bad about it
   2. If NO
      1. Probe
         1. Why haven’t you (or family member) used telehealth or remote medical visits (provider didn’t offer them, choose not to, didn’t have the equipment or internet needed)?
         2. What could your doctor or medical provider do to make you (or family member) more comfortable doing telehealth or remote visits?
2. Great thanks. I have some questions about the whole PREPARE NOW project and your work on the project in general. Please take a minute and think about your work on the PREPARE NOW project- starting from the beginning
   1. What was the main reason you decided to be part of the PREPARE NOW research project?
      1. LET THEM ANSWER- THEN
         1. What did you hope to gain from being part of the project?
            1. Did this happen?

YES or NO

Thanks, can you tell me more about this?

1. What was your favorite part of working on this PREPARE NOW project? (what have you enjoyed the most)
2. Can you tell me the top three things that this project did to help engage you, or help you participate in and work on this research project?
3. Did you feel that on the PREPARE NOW project, you were compensated (paid) appropriately for your time and expertise?
4. How much did you feel trust, honesty, transparency, shared-learning, and give-and-take relationships while working on this project?
   - 1. So, would you say:

☐ Not at all

☐ A little bit

☐ Somewhat

☐ A great deal

- - - - 1. **If [Somewhat OR A great deal]** Please provide an example of how you experienced trust, honesty, transparency, shared-learning, or give-and-take relationships

1. Sometimes there are challenges when researchers, patients, and other stakeholders work together. These might include finding a convenient time to meet or communicating clearly with each other. What have been the biggest challenges for you on this research project?
   - 1. What aspects of working on this project did you not like?
2. Can you tell me some things that the PREPARE NOW research project could have done differently to help you engage with, or participate in the project more?
3. Based on your experience with this research project, what would you suggest be done to help others contribute as research partners?
4. If the opportunity arose, would you be interested in working as a research partner on another research study?
   1. If NO
      1. Why would you not be interested in working as a research partner on another study?
5. Thanks so much for all of this great information, I just have one last question. Do you feel that you have had a meaningful impact (played an important role) on this PREPARE NOW research project?
   1. If YES
      1. Can you tell me more about that?
         1. How do you think you have had an important impact on the PREPARE NOW project?
         2. How did the researchers help make sure that you played an important role in this research project?
   2. If NOT SURE, DON’T KNOW, OR NO
      1. Can you tell me more about that?
         1. Why don’t you think you have had an important impact on the PREPARE NOW project?
         2. What could the researchers have done differently to help make sure that you played an important role in this research project?

**Great, those are all the questions I have for you. Is there anything else you would like to share with me about the PREPARE NOW project and your role in it?**

LET THEM ANSWER

**Thank you so much for the time you took with me today answering these questions, we really appreciate it. We will put everyone’s answers together into a paper and we will share it with you all next year. The information that you shared with us will help us do a better job on our research projects. This information will also help the community learn lessons from our project so that we can make future projects better for patients and their families.**

**Do you have any questions for me?**

**Thanks so much, have a great day!**

**INTERVIEWER FIELD NOTES ABOUT INTERVIEW (to be documented immediately after the interview):**

**Appendix B. Kidney Disease Organization Stakeholder Interview Guide**

**FILL OUT FOR EACH INTERVIEW:**

**Interview with (check and circle one, can cross check interviewee name with the list in Trello)**

- **Patient or family member Co-Investigator**
- **Kidney disease stakeholder**

**Assign the interview an identifier code:**

i.e. Co-I #1 (1-7), Stakeholder #1 (20-27)

**Hello! This is NAME calling from the PCORI PREPARE NOW study. Thanks so much for agreeing to talk to me today. We want to learn about how COVID-19 has impacted your life and also your ability to engage in our PCORI PREPARE NOW study. We would like to ask you some questions that should take less than an hour to complete.**

**Is this still a convenient time for you to talk?**

**If No**

No problem, we will reach out to you by email to schedule a better time to talk

*Email NAME and work with her to reschedule the interview*

**If Yes**

Great! I really appreciate your time today. I am going to go ahead and ask you some questions. Before I do, I want to assure you that what you share with me will be kept anonymous. I will record this interview just so I can be sure I don’t miss anything that you are saying- and the recording and transcript of our interview will be kept in protected and secure files. I am not going to ask your name, so your name will not be on the transcript or recording, and the information you give us will be combined with what other people tell us, so we will not be able to know who said what. We just want to talk to the people involved in the study to see how we did in engaging you in the research process, what are some good things we did, how we can make improvements- and also share lessons learned about how we can help patients, family members and stakeholders take part in research during COVID-19. If you have any questions, you can contact NAME at the University of South Carolina.

**Do you have any questions about anything I just said? Were there any parts that seemed unclear?**

**1. Do you agree to do this interview?**

- Yes
- No

**2. Do I have your permission to record this interview?**

- Yes
- No

**If no consent  Thank you for your time. Have a wonderful day.**

*****END INTERVIEW*****

**IF YES:** Thank you! **START RECORDING**

1. First, can you tell me- on this PREPARE NOW study team, were you a patient or family member Co-Investigator, or were you a kidney disease organization stakeholder?
2. I am going to start off by asking- in general, how has COVID-19 impacted the lives of the people with kidney disease you work with?
3. People with kidney disease are at high-risk for bad things to happen if they get COVID-19- do you think they have had to do things differently than other people who are in good health (not high-risk)?
   1. **Yes**
      1. What things have they had to do differently in their life because of COVID-19?
   2. **No**
      1. Great, thanks- what sorts of things have they been doing to be safe during COVID-19?
4. Thanks for sharing that. As you know, you have been part of the PCORI funded PREPARE NOW research study for the last five years as a kidney disease stakeholder. Can you think back starting earlier this year when COVID-19 started in the United State- until now- can you share with me if COVID-19 has made a difference (impacted/had an effect) in your participation and engagement with the PREPARE NOW project?
   1. (PROMPT-For example, were you unable to participate in meetings or read emails or information for this project because of COVID-19?)
   2. LET THEM ANSWER- THEN-
   3. **If YES effect**
      1. Thanks- can you tell me more about this
         1. LET THEM ANSWER- THEN
            1. **As you know, almost all of the PREPARE NOW project work you have done is done virtually- by phone, webinar or email. Has that been helpful for you to participate in the PREPARE NOW project during this year when COVID-19 was impacting everything?**

If yes- how so? Can you tell me more about that?

If no- thanks, can you tell me more about what you mean?

- 1. **If NO effect**
     1. Thanks- you started off mentioning some ways that COVID-19 has impacted your life- why do you think that it hasn’t impacted how you are able to participate and engage in the PREPARE NOW project?
        1. LET THEM ANSWER- THEN
           1. **As you know, almost all of the PREPARE NOW project work you have done is done virtually- by phone, webinar or email.** **Has that been helpful for you to participate in the PREPARE NOW project during this year when COVID-19 was impacting everything?**

If yes- how so? Can you tell me more about that?

If no- thanks, can you tell me more about what you mean?

1. Thanks so much. This is great information. Let me ask you- what are some ideas you have for how the PREPARE NOW team (or other research projects) can best help patient and family team members to fully engage in and be part of research projects while dealing with COVID-19’s impact on themselves and their families?
   1. Probe for specific suggestions- can also restate: We know that COVID-19 is going to be around for awhile, what are the best ways for researchers to partner with patients and family members in research during COVID-19?
      1. LET THEM ANSWER- THEN
         1. As you know, almost all of the PREPARE NOW project work is done virtually, or remotely- by phone, webinar or email. What might be some issues or problems with that for other patients and family members working with different research projects? Why might that be a problem?
            1. LET THEM ANSWER- THEN

Thanks, what are some things that our project has done well to keep you engaged and active, that we can implement on other research projects where we can involve stakeholders on research teams during COVID-19?

1. What has your experience been (what has it been like for you) with our virtual form of research engagement? (work done remotely, through emails, calls and webinar meetings)
   1. Probe for specific suggestions
      1. Do you like doing work this way?
      2. What are the best things about it?
      3. What don’t you like about it?
      4. What would you like done differently?
2. Overall, how satisfied and engaged have you felt working on the PREPARE NOW project as a stakeholder?
   1. LET THEM ANSWER- THEN
      1. Has this changed- how satisfied and engaged you have felt- before and after COVID-19?
         1. If **NO**
            1. Thanks, why do you think you haven’t felt any differently about how satisfied you are with your work as a stakeholder on the PREPARE NOW project? can you describe ways your engagement on this project has NOT been affected by COVID-19?
            2. Additional probes- **some people may feel that being part of a research project during COVID-19 could offer hope or positivity in this difficult time, or some others may feel it adds additional stress. Have you felt this way being part of PREPARE NOW or any other research projects during COVID-19?**
         2. If **YES**
            1. Thanks, please tell me a little bit more about how COVID-19 changed the way you feel satisfied and engaged with your work as a stakeholder on the PREPARE NOW project?- can you describe ways your engagement on this project has been affected by COVID-19?

Additional probes- **some people may feel that being part of a research project during COVID-19 could offer hope or positivity in this difficult time, or some others may feel it adds additional stress. Have you felt this way being part of PREPARE NOW or any other research projects during COVID-19?**

- 1. Do you have any suggestions about how the research team can best help organizations engage in research during the COVID-19 pandemic?

1. Have you heard about people with kidney disease missing any medical appointments, or not getting any bloodwork or medical testing done, because of COVID-19?
   1. If YES
      1. Probe- can you tell me more about that? what have you heard?
         1. What do you think doctors and healthcare providers can do that would make people with kidney disease feel better about going to medical appointments during COVID-19? (What can they do to help patients feel safer?)
2. Have you heard about people with kidney disease using telehealth or remote medical visits (by phone, or the internet) because of COVID-19?
   1. If YES
      1. Probe—can you tell me more about that?
         1. What appointments have they done using telehealth?
         2. What was their experience been using telehealth or remote visits?
3. Great thanks. I have some questions about the whole PREPARE NOW project and your work on the project in general. Please take a minute and think about your work on the PREPARE NOW project—starting from the beginning
   1. What was the main reason you decided to be part of the PREPARE NOW research project?
      1. LET THEM ANSWER—THEN
         1. What did you hope to gain from being part of the project?
            1. Did this happen?

If YES or NO

Thanks, can you tell me more about this?

1. What was your favorite part of working on this PREPARE NOW project? (what have you enjoyed the most)
2. Can you tell me the top three things that this project did to help engage you, or help you participate in and work on this research project?
3. Did you feel that on the PREPARE NOW project, you were compensated (paid) appropriately for your time and expertise?
4. How much did you feel trust, honesty, transparency, shared-learning, and give-and-take relationships while working on this project?
   - - 1. So, would you say:
          1. ☐ Not at all
          2. ☐ A little bit
          3. ☐ Somewhat
          4. ☐ A great deal

**If [Somewhat OR A great deal]** Please provide an example of how you experienced trust, honesty, transparency, shared-learning, or give-and-take relationships

1. Sometimes there are challenges when researchers, patients, and other stakeholders work together. These might include finding a convenient time to meet or communicating clearly with each other. What have been the biggest challenges for you on this research project?
   - 1. What aspects of working on this project did you not like?
2. Can you tell me some things that the PREPARE NOW research project could have done differently to help you engage with, or participate in the project more?
3. Based on your experience with this research project, what would you suggest be done to help others contribute as research partners?
   1. If the opportunity arose, would you be interested in working as a research partner on another research study?
      1. If NO
         1. Why would you not be interested in working as a research partner on another study?
4. So, do you have any thoughts about how the research team can best help organizations engage during the pandemic?
5. Thanks so much for all of this great information, I just have one last question. Do you feel that you have had a meaningful impact (played an important role) on this PREPARE NOW research project?
   1. If YES
      1. Can you tell me more about that?
         1. How do you think you have had an important impact on the PREPARE NOW project?
         2. How did the researchers help make sure that you played an important role in this research project?
   2. If NOT SURE, DON’T KNOW, OR NO
      1. Can you tell me more about that?
         1. Why don’t you think you have had an important impact on the PREPARE NOW project?
         2. What could the researchers have done differently to help make sure that you played an important role in this research project?

**Great, those are all the questions I have for you. Is there anything else you would like to share with me about the PREPARE NOW project and your role in it?**

LET THEM ANSWER

**Thank you so much for the time you took with me today answering these questions, we really appreciate it. We will put everyone’s answers together into a paper and we will share it with you all next year. The information that you shared with us will help us do a better job on our research projects. This information will also help the community learn lessons from our project so that we can make future projects better for patients and their families.**

**Do you have any questions for me?**

**Thanks so much, have a great day!**

**INTERVIEWER FIELD NOTES ABOUT INTERVIEW (to be documented immediately after the interview):**
